# Nutrition and health canteen-based lifestyle intervention: association with weight management, lipid metabolism regulation, and inflammation alleviation in middle-aged adults

**DOI:** 10.3389/fnut.2026.1754647

**Published:** 2026-04-01

**Authors:** Xiaoqin Luo, Xiaoxiao Guo, Haige Cao, Dan Cao, Guohua Li, Xinyue Wang, Jun Liu, Yimei Du, Yuexin Yang

**Affiliations:** 1 Department of Nutrition and Food Safety, School of Public Health, Xi’an Jiaotong University, Xi’an, China; 2 Key Laboratory for Disease Prevention and Control and Health Promotion of Shaanxi Province, Xi’an, China; 3 Xi’an No. 3 Hospital, The Affiliated Hospital of Northwest University, Xi’an, China; 4 School of Sports and Health Science, Xi’an Physical Education University, Xi’an, China; 5 Department of Clinical Nutrition, Xi’an Chest Hospital, Xi’an, China; 6 National Institute for Nutrition and Health, Chinese Center for Disease Control and Prevention, Beijing, China

**Keywords:** body weight, health education, lipid metabolism, non-communicable diseases, nutrition and health canteen

## Abstract

**Background:**

Lifestyle interventions can prevent and manage non-communicable diseases (NCDs); however, they are not easy to implement in daily life. Nutrition policy-based interventions with both high impact and feasibility are needed for NCD prevention and control.

**Methods:**

This single-group before–after study was conducted for 12 weeks in a collective catering setting, where the construction of a nutrition and health canteen, proposed by the *Reasonable Diet Action*, is currently underway in China. The intervention for participants comprised a multifaceted approach featuring rich food diversity, a reduced supply of salt/sugar/oil, and structured health education on both diet and physical activity. The primary outcome was the change in body weight after the intervention. Demographic data, food frequency questionnaire results, and anthropometric data were also collected. Blood biochemical indicators and inflammatory biomarkers were also measured.

**Results:**

A total of 97 participants (33 women) were included in this study. Of the 43 individuals (44.3%) who were overweight, 8 (8.4%) were obese. After the intervention, the body weight of participants significantly decreased [74.00 (61.05–81.6) kg vs. 72.35 (58.83–80.15) kg, *p* < 0.001]. The secondary outcomes also showed a trend toward benefit, including body mass index (BMI) and waist-to-hip ratio (*p* < 0.05). In particular, indicators of lipid metabolism, such as triglycerides [1.17 (0.78–1.70) mmol/L vs. 1.12 (0.74–1.87) mmol/L, *p*_FDR_ = 0.047], low-density lipoprotein cholesterol (LDL-C, 2.76 ± 0.70 mmol/L vs. 2.58 ± 0.62 mmol/L, *p*_FDR_ = 0.041), and apolipoprotein B (0.83 ± 0.20 g/L vs. 0.78 ± 0.18 g/L, *p*_FDR_ = 0.045) slightly decreased, whereas HDL cholesterol (1.32 ± 0.33 mmol/L vs. 1.37 ± 0.34 mmol/L, *p*_FDR_ = 0.044) increased. However, total cholesterol, blood pressure, insulin, glucose, and HOMA-IR did not differ after the intervention. Notably, the levels of inflammatory biomarkers, IL-6, IL-8, TNF-α, and hs-CRP decreased markedly after the intervention.

**Conclusion:**

The nutrition and health canteen strategy may be a feasible approach for implementing lifestyle interventions to promote body weight control, improve lipid metabolism, and reduce inflammation in middle-aged adults. Further studies, especially randomized controlled trials with longer terms, are warranted.

## Introduction

The prevalence and mortality of non-communicable diseases (NCDs), including cardiovascular and cerebrovascular diseases (CVDs), metabolic syndrome (MetS), type 2 diabetes mellitus (T2DM), and cancers, are increasing, especially in China (1, 2). Unhealthy lifestyles are the main causes of NCDs, such as unhealthy dietary habits and physical inactivity (3, 4). Therefore, an increasing number of studies have focused on lifestyle interventions to prevent and control NCDs, especially through diet and exercise. Recently, DeWalt et al. (5) proposed a simple categorization scheme for potential proactive prevention interventions, “graded” along two dimensions: impact and feasibility. Theoretically, dietary approaches are among the most compelling strategies for reducing disability and death. For instance, studies have found that the Chinese heart-healthy (CHH) diet (6, 7) and the dietary approaches to stop hypertension (DASH) pattern are both promising and effective for hypertension prevention (8). However, these dietary interventions are usually high-impact but low-feasibility and they are not easy to implement and actively adhere to in daily life because people are always required to obey a relatively strict dietary recommendation (9).

Rather than encouraging individuals to change their lifestyle or eating habits, other proactive approaches, such as modifying the diet in collective catering settings, are more likely to achieve satisfactory results and should be prioritized for implementation (10, 11). Recently, in China, a series of nutrition and public health policies, such as the “Healthy China Initiative 2030” (12) and the “National Nutrition Plan of China (2017–2030)” (13), have been launched to prevent the increasing incidence of NCDs. Specifically, the construction of nutrition and health canteen proposed by the *Reasonable Diet Action* is to implement the relevant requirements of these nutrition policies and respond to the call of “formulating and implementing the nutritional operation standards of collective catering and carrying out the creation activities of demonstration healthy canteens and restaurants” (14). The purpose of this strategy is to promote healthy cooking practices (e.g., “three reductions” strategy and balanced meals), improve nutrition and health awareness of catering providers, and cultivate residents’ healthy dietary habits. Among the numerous detailed rules and requirements in the construction of nutrition and health canteens, enriching food diversity and reducing the supply of salt, sugar, and oil, which contribute to a large proportion of NCD cases, are the easiest to implement (15). In addition, health education is also recommended, as individuals with low health literacy face a 1.5–3 times higher risk of these adverse health outcomes compared to those with high health literacy (5, 16), and the importance of health education is well established (17–19). Recently, a randomized controlled trial supported the application of personalized dietary advice for cardiometabolic health (20). Hence, China’s collective dining system in government departments, enterprises, schools, factories, and other places is supposed to provide unique advantages and feasibility for health education and promotion to prevent NCDs. To date, little is known about the role of nutrition and health canteen in improving adult metabolism.

Therefore, we hypothesized that a multilevel approach based on nutrition and health canteen strategy would improve the efficacy of advice in eliciting meaningful health outcomes. A 12-week single-group pre-post pilot study was designed to assess a dietary intervention combined with structured dietary and exercise advice on metabolic outcomes in a generally representative group of middle-aged adults.

## Methods

### Study design and participants

This single-group pre-post pilot study was conducted at an administrative training school that provided centralized accommodation and dining in Xi’an, China. The eligible participants were the first batch of trainees in 2022 who were willing to participate in the study. Individuals were excluded if they had serious diseases, such as cancer, CVD, liver and kidney dysfunction, serious active infection, severe cognitive or mental impairment, a history of trauma or surgery in the month before the study, any other contraindications for physical activities, weight loss in the past 3 months due to vomiting or medication, or any form of participation in a similar intervention program. Pregnant or lactating women were also excluded. Data, including demographic data, food frequency questionnaire, anthropometric data, and blood samples, was collected 1 week before the intervention and completed within 1 week after the intervention.

This study was approved by the Medical and Biological Research Ethics Committee of Xi’an Jiaotong University (2022-524). The entire research process was conducted in accordance with the basic principles of the Helsinki Declaration. Written informed consent was obtained from all participants.

### Interventions

#### Dietary intervention

For 12 weeks, participants’ meals were collectively served by the canteen, which implemented a “three reduction” strategy, including the reduction of the consumption of salt (less than 6 g daily per person), sugar (less than 50 g daily per person), and oil (less than 30 g daily per person). Each dish was specially equipped with a nutrition label marking the composition and content of the nutrients contained in the food. The participants were required to have concentrated meals in the canteen at least from Monday to Friday during the intervention period, and the meals on the weekend followed structured nutrition and health education.

#### Health education

Health education focused on both diet and physical activity. At the beginning of the intervention, an expert delivered a two-hour lecture on centralized nutrition and health to all participants. The information conveyed in the lecture included the meaning of proper nutrition and its relationship with human health; how to choose food with the help of the Chinese Balanced Diet Pagoda (CBDP) (2022); the importance and practice of nutrition labels; the criteria for judging healthy weight; and the potential harm caused by obesity and NCDs. During the intervention period, an online nutrition health education program with systematic and specific health education content was weekly pushed to the participants through WeChat (a chat software) management (Supplementary Table 1). All the participants were encouraged to take photos of their meals and send them to well-trained registered dietitians for advanced dietary suggestions. The dietitians were also responsible for providing dietary health guidance on weekends and public holidays. A similar process was carried out for physical activity health education. In general, two fitness coaches introduced structured exercises in the WeChat group every week and encouraged participants to attend at least 1 h of exercise three times a week. The specific cycle training tasks, objectives, methods, etc., are shown in Supplementary Table 2.

In addition, the canteen was equipped with a supportive environment, which is one of the construction requirements for a nutrition and health canteen. For example, a professional weighing scale was installed in the canteen for everyone to use, and posters promoting “three reductions” and the dietary pagoda were prominently placed. Simultaneously, in response to sports health education and guidance, conventional sports equipment and venues were available to all participants.

### Data collection

#### Dietary and exercise assessment

A Food Frequency Questionnaire (FFQ) was used to calculate food intake before the intervention. During the intervention, the daily per capita consumption of various foods = (amount purchased − the residual)/(person per day) from each Monday to Friday was recorded. The participants were encouraged to select foods in accordance with dietary guidelines and provide their dietary check-in records in the WeChat group on weekends and public holidays. Nutrient intake was calculated according to the China Food Composition Table Volume 1, Edition 2. Since total energy intake may have a potential effect on nutrients, the residual method was used when analyzing changes in dietary nutrients before and after the intervention. Specifically, we first regressed each nutrient intake (dependent variable) on total energy intake (independent variable) to obtain residual values. The residuals were used for subsequent statistical analyses to reflect the relative nutrient composition of the diet rather than absolute intake. For exercise, the participants completed the International Physical Activity Questionnaire (IPAQ, short form) to assess daily exercise frequency, duration, and type at baseline.

#### Physical examination

All participants underwent a physical examination after overnight fasting before and after the intervention. Height, weight, and waist–hip circumference were measured in accordance with the “WS/T424-2013 Anthropometric method for Population Health Monitoring” standard in the morning during fasting, and body mass index (BMI) and Waist-to-Hip Ratio (WHR) were calculated. Body composition was measured using an InBody160 body meter (China Medical Device Import Registration: 20172210600). After resting in a quiet environment for 5–10 min, the left brachial artery blood pressure was measured using a mercury-standard cuff sphygmomanometer. Well-trained research assistants were responsible for collecting anthropometric data and blood pressure measurements. Three measurements were taken, and the mean of the last two measurements was used for the analyses.

### Laboratory test

Fasting peripheral venous EDTA blood samples were collected from all participants after overnight fasting and shipped in dry ice to the Xi’an Chest Hospital and stored at −80 °C in aliquots until analysis. All blood indicators were tested twice in parallel, and the average value was used as the final result. Fasting blood glucose (FBG), total cholesterol (TC), HDL cholesterol (HDL-C), LDL cholesterol (LDL-C), apolipoprotein A1 (ApoA1), apolipoprotein B (ApoB), and triglycerides (TG) levels were measured enzymatically using an automatic analyzer (Hitachi 7080, Tokyo, Japan). Insulin was measured using an AntuLumo A2000 Plus fully automated chemiluminescence immunoassay analyzer. Insulin and FBG levels were used to calculate the homeostasis model assessment of insulin resistance (HOMA-IR): HOMA-IR = FBG (mmol/L) × insulin (μIU/mL)/22.5. High-sensitivity concentration of C-reactive protein (hs-CRP) in blood was measured using Mindray BC-5390CRP latex immunoturbidimetric technology. Interleukin-6 (IL-6) was measured using a Snibe MAGLUMI X8 fully automated chemiluminescence immunoassay system. Interleukin-8 (IL-8) and tumor necrosis factor α (TNF-α) were measured using a sandwich ELISA assay (R&D Systems, Minneapolis, MN).

### Statistical analysis

Measurement data are expressed as the mean ± standard deviation if they follow a normal distribution and in the form of a median with an interquartile range (IQR) if they are skewed. Counting data are expressed as counts (percentages). For pre- and post-intervention comparisons, paired t-tests were used for normally distributed data, and Wilcoxon signed-rank tests were used for non-normally distributed data. Categorical variables were tested using the chi-square test. Multiple comparison adjustments were performed using the false-discovery rate (FDR). In this study, the Statistical Package for Social Sciences (SPSS version 26.0) was used for data analysis, and a bilateral test was used. A *p*-value of <0.05 was considered statistically significant.

## Results

### Baseline characteristics

**Figure 1:**
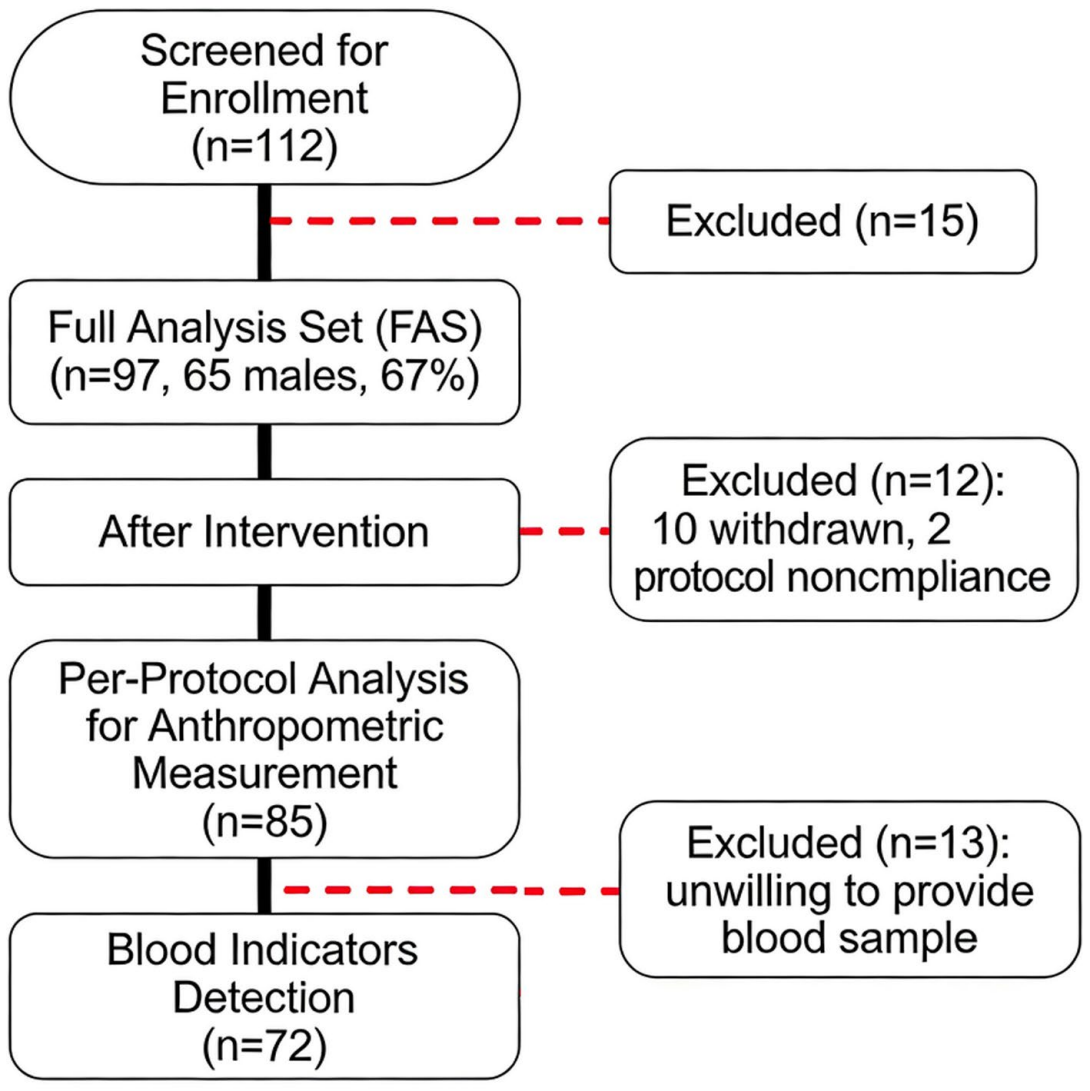
Flowchart of participants’ enrollment. Flowchart depicting the procedures of participant recruitment, eligibility screening, and intervention in this study. A total of 112 participants were screened, and 97 were enrolled, with 10 withdrawn and 2 protocol non-compliance during intervention. Another 13 participants were not willing to provide a blood sample after the intervention.

Overall, of the 112 participants screened for enrollment in the study, 97 (65 men, 67%) were included in the full analysis set. After the intervention, 85 were included in the per-protocol analysis for anthropometric measurement (10 were withdrawn and 2 were protocol non-compliant), and 72 were included in blood indicator detection (another 13 were not willing to provide a blood sample after the intervention) (Figure 1). As shown in Table 1, the median age of all participants was 43 (39–48.5) years. They had a median weight of 74 (61.05–81.6) kg and WHR of 0.89 (0.85–0.91), with 43 (44.3%, 39 men) individuals overweight and 8 (8.2%, 8 men) obese. In terms of blood pressure, glucose, and lipid metabolism, 15 (15.9%) individuals had SBP ≥130 mmHg, 27 (28.7%) had DBP ≥80 mmHg; 4 (4.1%) had FBG >6.1 mmol/L; 6 (6.2%) had TC ≥6.2 mmol/L, 13 (13.4%) had HDL-C <1 mmol/L, 3 (3.1%) had LDL-C ≥4.1 mmol/L, and 44 (45.4%) had TG ≥1.7 mmol/L. Besides, the prevalence of NCDs in men was much higher than that in women: obesity (12.3% vs. 0), hypertension (38.7% vs. 9.4%), hyperlipidemia (49.2% vs. 37.5%), prediabetes (4.6% vs. 3.1%), and hypercholesterolemia (9.2% vs. 0). All participants had a relatively high education, a bachelor’s degree or above. More than half of the participants exercised only 1–2 times per week before the intervention, with relatively short duration and low intensity (Supplementary Table 3).

**Table 1:** Baseline characteristics of participants.

	Men	Women	Total
Number of people (%)	65 (67.0)	32 (33.0)	97 (100)
Age (years)	43 (39–49.5)	40.5 (38–46.75)	43 (39–48.5)
Education (%)			
Undergraduate course	20 (30.8)	7 (21.9)	27 (27.8)
Master	29 (44.6)	16 (50.0)	45 (46.4)
Doctor	16 (24.6)	9 (28.1)	25 (25.8)
Height (cm)	175.50 ± 5.29	165.97 ± 4.60	172.36 ± 6.76
Weight (kg)	78.3 (73.95–83.55)	58.2 (52.98–65.28)	74 (61.05–81.6)
BMI (kg/m^2^) (%)			
<18.5	1 (1.5)	5 (15.6)	6 (6.2)
18.5 ≤ BMI < 24.0	17 (26.2)	23 (71.9)	40 (41.2)
24.0 ≤ BMI < 28.0	39 (60.0)	4 (12.5)	43 (44.3)
≥28.0	8 (12.3)	0 (0)	8 (8.3)
WHR	0.90 (0.88–0.92)	0.84 (0.80–0.87)	0.89 (0.85–0.91)
High WHR (%)^a^	45.3	73.3	55.4
Body fat (kg)	19.04 ± 4.56	16.58 ± 5.99	18.16 ± 5.22
Body fat level (%)^b^			
Low	1 (1.9)	4 (13.3)	5 (6.0)
Normal	4 (7.5)	6 (20.0)	10 (12.1)
High	48 (90.6)	20 (66.7)	68 (81.9)
Pulse (%)			
<60 times/min	4 (6.5)	0 (0)	4 (4.3)
60–100 times/min	56 (90.3)	31 (96.9)	87 (92.5)
>100 times/min	2 (3.2)	1 (3.1)	3 (3.2)
SBP (%)^c^			
<90 mmHg	1 (1.6)	0 (0)	1 (1.1)
90–130 mmHg	48 (77.4)	30 (93.8)	78 (83.0)
≥130 mmHg	13 (21.0)	2 (6.2)	15 (15.9)
DBP (%)^c^			
<60 mmHg	5 (8.1)	9 (28.1)	14 (14.9)
60–80 mmHg	33 (53.2)	20 (62.5)	53 (56.4)
≥80 mmHg	24 (38.7)	3 (9.4)	27 (28.7)
FBG (%)^c^			
<3.9 mmol/L	0 (0)	0 (0)	0 (0)
3.9–6.1 mmol/L	62 (95.4)	31 (96.9)	93 (95.9)
>6.1 mmol/L	3 (4.6)	1 (3.1)	4 (4.1)
TC (%)^c^			
<6.2 mmol/L	59 (90.8)	32 (100)	91 (93.8)
≥6.2 mmol/L	6 (9.2)	0 (0)	6 (6.2)
HDL-C (%)^c^			
<1.0 mmol/L	13 (20.0)	0 (0)	13 (13.4)
≥1.0 mmol/L	52 (80.0)	32 (100)	84 (86.6)
LDL-C (%)^c^			
<4.1 mmol/L	62 (95.4)	32 (100)	94 (96.9)
≥4.1 mmol/L	3 (4.6)	0 (0)	3 (3.1)
TG (%)^c^			
<1.7 mmol/L	33 (50.8)	20 (63.5)	53 (54.6)
≥1.7 mmol/L	32 (49.2)	12 (37.5)	44 (45.4)

a High WHR definition: men ≥0.9 and women ≥0.8.

b Body fat levels are classified according to the normal body fat percentage of men, ranging from 15 to 18%, and women, ranging from 20 to 25%. Values below the lower limit of the reference range were identified as a low body fat level, and those above the upper limit were identified as a high body fat level.

c Grouping based on the diagnostic criteria for metabolic syndrome.

### Dietary intake at baseline and during intervention

During the 12-week intervention period, a total of 21,015 person-times were spent dining. The data on the total purchase and residual quantity of various foods were collected from the Food and Beverage Department and displayed in Supplementary Table 4. The composition of the participants’ habitual diets at baseline and dietary intake during the intervention is shown in Table 2. After the intervention, participants reduced the consumption of oil (37.05 vs. 29.27 g, *p* < 0.001) and salt (7.38 vs. 5.89 g, *p* < 0.001), while having comparable sugar intake (26.33 vs. 27.13 g, *p* > 0.05). They decreased the consumption of rice, wheat, and fried food but increased that of fresh vegetables, fruits, fish, other aquatic products, eggs, milk, and yogurt (all *p* < 0.05). They also had a decent change in energy intake from baseline, with 1655.40 (1371.99–1995.75) kcal per day vs. 1438.30 kcal per day (*p* < 0.001). For macronutrients, the intake of protein increased [56.10 (48.66–64.83) g vs. 58.00 g, *p* = 0.043] while that of carbohydrate decreased [260.42 (228.17–283.93) g vs. 250.40 g, *p* = 0.028], making a similar change in their energy supply ratio (*p* < 0.001). The intake of dietary fiber grew significantly from 9.06 g to 11.74 g (*p* < 0.001). The mean 12-week micronutrient intakes were 10.26 (6.73–14.55) mg vs. 14.36 mg for vitamin E, 10.26 (6.73–14.55) mg vs. 0.79 mg for vitamin B1, 0.73 (0.61–0.84) mg vs. 0.79 mg for vitamin B2, 12.06 (9.72–14.95) mg vs. 13.20 mg for vitamin B3, and 68.72 (44.37–95.32) mg vs. 79.50 mg for vitamin C (all *p* < 0.001).

**Table 2:** Dietary intake at baseline and average daily dietary intake during the intervention (*n* = 85).

	Before intervention	During intervention	*p*-value
Food consumption			
Oil (g)	37.05 (23.40–45.26)	29.27	**<0.001**
Salt (g)	7.38 (5.04–10.11)	5.89	**<0.001**
Sugar (g)	25.83 (19.06–29.55)	27.13	0.527
Rice (g)	75.33 ± 12.09	69.76	**<0.001**
Wheat (g)	123.59 ± 23.35	103.41	**0.043**
Coarse cereals (g)	58.88 ± 0.43	62.86	0.682
Potato (g)	18.16 ± 5.22	17.84	0.953
Fresh vegetable (g)	126.48 ± 5.30	155.80	**0.032**
Fresh fruit (g)	108.02 ± 5.59	127.25	**0.043**
Pork (g)	57.17 ± 6.88	62.32	0.055
Red meat (g)	45.99 ± 6.63	52.98	0.056
Poultry (g)	36.92 ± 6.68	36.09	0.782
Fish (g)	19.94 ± 1.85	22.69	**<0.001**
Other aquatic products (g)	10.32 ± 1.78	15.67	**<0.001**
Eggs (g)	43.46 ± 0.59	50.38	**0.006**
Milk (g)	77.98 ± 11.85	100.02	**<0.001**
Yoghurt (g)	114.30 ± 14.22	133.34	**<0.001**
Soya bean (g)	7.98 ± 1.85	8.02	0.326
Soybean products (g)	17.72 ± 1.62	19.02	0.224
Nuts (g)	7.91 ± 1.63	8.38	0.190
Fried food (g)	114.30 ± 14.22	78.34	**0.035**
Dietary nutrients^a^			
Total energy (kcal)	1655.40 (1371.99–1995.75)	1438.30	**<0.001**
Protein (%)	14.38 (10.78–18.21)	16.64	**<0.001**
Fat (%)	17.78 (11.18–24.88)	21.75	**<0.001**
Carbohydrate (%)	68.25 (50.88–90.72)	62.69	**<0.001**
Protein (g)	56.10 (48.66–64.83)	58.00	**0.043**
Fat (g)	30.85 (19.89–43.56)	32.93	0.057
Carbohydrate (g)	260.42 (228.17–283.93)	250.40	**0.028**
Dietary fiber (g)	9.06 (7.09–11.52)	11.74	**<0.001**
Vitamin A (μgRE)	310.33 (197.96–491.43)	291.65	0.104
Vitamin E (mg)	10.26 (6.73–14.55)	14.36	**<0.001**
Vitamin B1 (mg)	0.67 (0.54–0.81)	0.79	**<0.001**
Vitamin B2 (mg)	0.73 (0.61–0.84)	0.79	**<0.001**
Vitamin B3 (mg)	12.06 (9.72–14.95)	13.20	**<0.001**
Vitamin C (mg)	68.72 (44.37–95.32)	79.50	**<0.001**

a Calculate nutrient intake for energy adjustment. The bold values indicate statistical significance (*p* < 0.05) compared to baseline.

### Effects of intervention on anthropometry and metabolism

**Figure 2:**
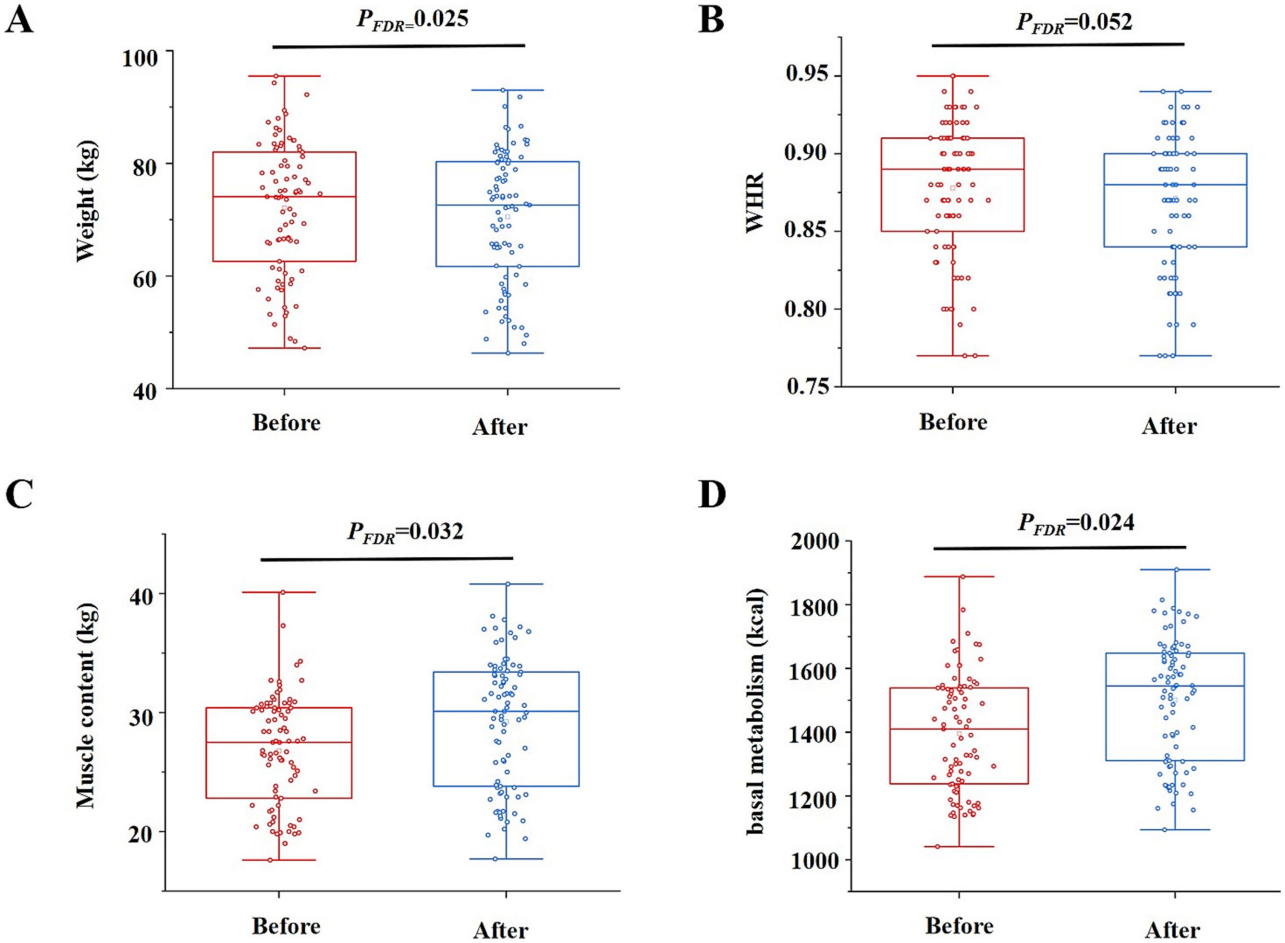
Changes in median levels of body weight (*p*_FDR_ = 0.025) **(A)**, waist-to-hip ratio (WHR, *p*_FDR_ = 0.013) **(B)**, muscle content (*p*_FDR_ = 0.001) **(C)**, and basal metabolism (*p*_FDR_ = 0.003) **(D)** in participants before and after the intervention (*n* = 85).

As shown in Figure 2, the body weight [74.00 (61.05–81.6) kg vs. 72.35 (58.83–80.15) kg, *p*_FDR_ = 0.025] and BMI (23.86 ± 3.14 kg/m^2^ vs. 23.37 ± 3.18 kg/m^2^, *p*_FDR_ = 0.016) of participants significantly decreased, whereas WHR (0.89 (0.85–0.91) vs. 0.87 (0.82–0.9), *p*_FDR_ = 0.052) remained comparable with those before the intervention. We also assessed participants’ body composition and observed an increase in muscle content and basal metabolism, along with a reduction in body water content, protein, and inorganic salt levels. Body fat mass and blood pressure remained unchanged (Supplementary Table 5).

**Figure 3:**
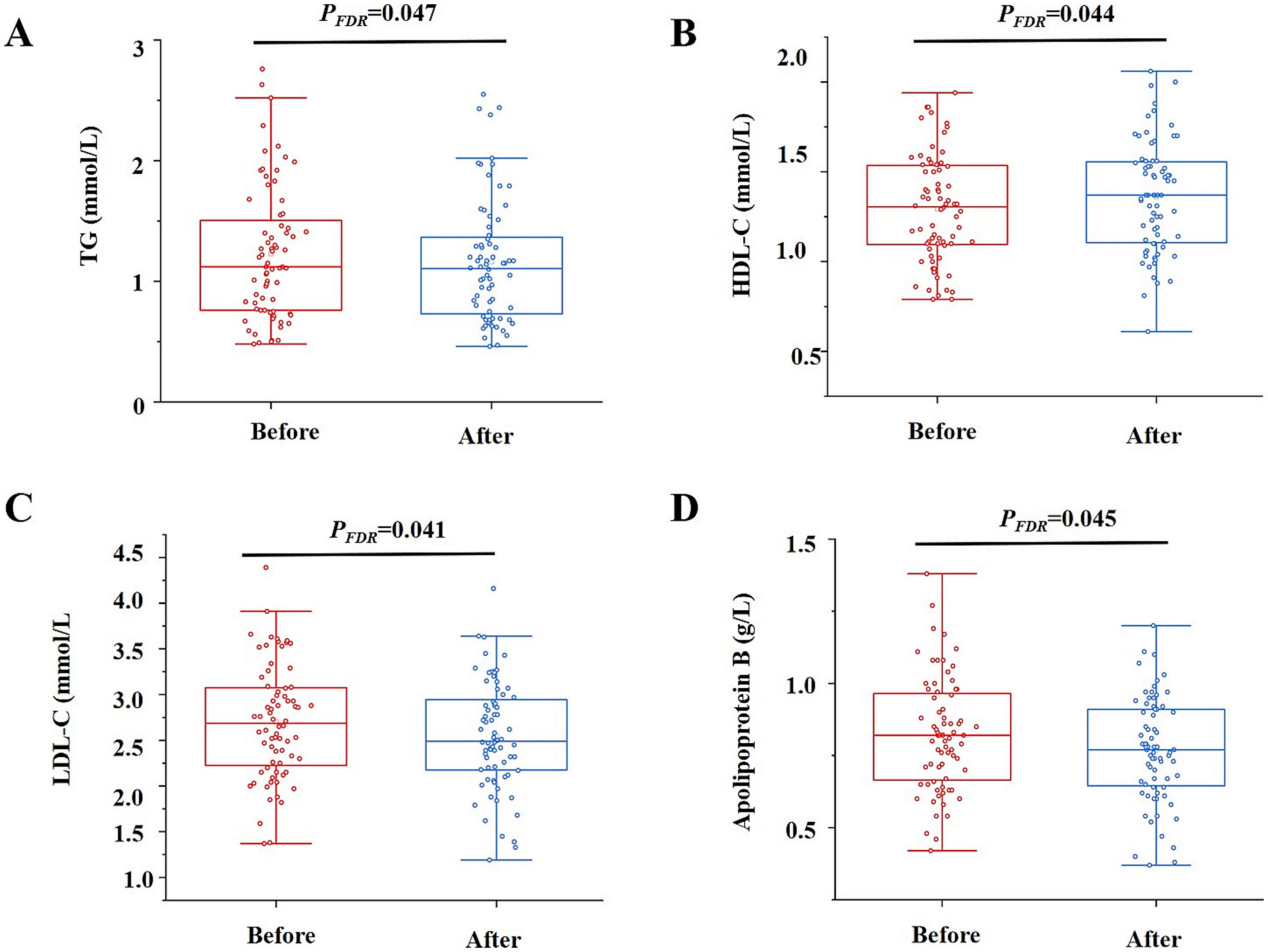
Effects of intervention on blood lipid metabolism. Primary analysis of the effects of the intervention on lipid metabolism. Data shown are median levels of serum TG **(A)**; mean differences of HDL-C **(B)**, LDL-C **(C)**, and apolipoprotein B **(D)** in participants before and after the intervention (*n* = 72). TG, triglycerides; HDL-C, high-density lipoprotein cholesterol; LDL-C, low-density lipoprotein cholesterol.

The effects of intervention on metabolism are shown in Figure 3. The level of insulin [8.29 (6.69–10.71) μIU/mL vs. 9.18 (6.86–11.90) μIU/mL, *p*_FDR_ = 0.092] slightly but not significantly increased, and no obvious change of FBG [4.96 (4.66–5.29) mmol/L vs. 4.76 (4.48–5.13) mmol/L] and HOMA-IR [(1.98 ± 1.16) vs. (2.29 ± 1.56)] was found. In terms of blood lipid profile, the level of TG [1.17 (0.78–1.70) mmol/L vs. 1.12 (0.74–1.87) mmol/L, *p*_FDR_ = 0.047], LDL-C (2.76 ± 0.70 mmol/L vs. 2.58 ± 0.62 mmol/L, *p*_FDR_ = 0.041), and apolipoprotein B [(0.83 ± 0.20) g/L vs. (0.78 ± 0.18) g/L, *p*_FDR_ = 0.045] of participants marginally decreased after the intervention, whereas the HDL-C [(1.32 ± 0.33) mmol/L vs. (1.37 ± 0.34) mmol/L, *p*_FDR_ = 0.044] level marginally increased. Differences in TC [(4.90 ± 0.80) vs. (4.80 ± 0.79), *p*_FDR_ = 0.103] concentrations were not statistically significant (Supplementary Table 5). The changes in inflammatory biomarkers before and after the intervention were significantly variable (Table 3). Compared to the baseline, the levels of IL-6 [76.67 (32.78–141.63) pg/mL vs. 28.29 (8.78–79.53) pg/mL, *p*_FDR_ = 0.015], IL-8 [102.03 (89.25–110.25) pg/mL vs. 39.43 (29.38–44.51) pg/mL, *p*_FDR_ = 0.009], TNF-α [28.22 (22.84–30.76) pg/mL vs. 12.33 (10.49–13.75) pg/mL, *p*_FDR_ = 0.031], and hs-CRP [0.58 (0.28–1.22) mg/L vs. 0.15 (0.10–0.75) mg/L, *p*_FDR_ = 0.027] of participants dramatically dropped after the intervention. No adverse events were reported during the whole study.

**Table 3:** Effects of intervention on inflammatory biomarkers.

Inflammatory biomarker	Before intervention	During intervention	*p*-value	*p*_FDR_ ^a^
IL-6 (pg/mL)	76.67 (32.78–141.63)	28.29 (8.78–79.53)	<0.001	0.015
IL-8 (pg/mL)	102.03 (89.25–110.25)	39.43 (29.38–44.51)	<0.001	0.009
TNF-α (pg/mL)	28.22 (22.84–30.76)	12.33 (10.49–13.75)	<0.001	0.031
hs-CRP (mg/L)	0.58 (0.28–1.22)	0.15 (0.10–0.75)	0.009	0.027

Data shown are median and interquartile range (IQR) of circulating IL-6, IL-8, TNF-α and hs-CRP in participants before and after the intervention (*n* = 72). IL-6, interleukin-6; IL-8: interleukin-8; TNF-α, tumor necrosis factor α; hs-CRP, high-sensitivity C-reactive protein.

a *p*_FDR_ means the *p*-value after multiple comparisons using the false discovery rate (FDR) method.

## Discussion

In this 12-week single-group pre-post pilot study, a nutrition and health canteen strategy-based lifestyle intervention, predominantly through a diet and exercise approach, resulted in great improvements in diet quality and compelling changes in the metabolism of adults, as indicated by favorable reductions in body weight, BMI, TG/LDL-C/apolipoprotein B concentrations, and inflammatory biomarker (IL-6, IL-8, TNF-α, and hs-CRP) levels.

Our findings support the implementation of this nutrition and health canteen strategy-based lifestyle intervention to improve body weight. Many countries worldwide have released national dietary guidelines but have not yet implemented cost-effective policies to put them into practice. In contrast to previous studies, in response to the implementation of nutrition and health canteen construction proposed by the *Reasonable Diet Action*, we conducted interventions in an administrative training school, which has a centralized accommodation system and is constructing a nutrition-based healthy canteen. This provided us with an opportunity to prevent and control NCDs in settings closer to real life. In this study, the participants can represent middle-aged people with relatively high levels of education in China. The prevalence of NCDs in this population, such as overweight/obese, hyperglycemia, and hyperlipemia, was similar to that in the general adult population (21). Increased food diversity, reduced supply of salt, sugar, and oil, along with structured health education resulted in significant improvements in body weight and composition. Our findings are consistent with a previous study in which structured or personalized lifestyle approaches led to benefits in weight loss or improved metabolism (22). Although the weight loss observed in our study was moderate, it was still associated with health outcomes, which were consistent with a previous study (23). The results for body composition supported the effectiveness of the intervention. Although the fat mass was not reduced after the intervention, the favorable effects on muscle content, body moisture content, and basal metabolism may reflect the impact of multiple levels of approaches on participants. Our findings indicate that this nutrition and health canteen strategy-based lifestyle intervention makes body weight control effective and feasible.

In terms of improving metabolism, the most impressive change was a decline in systemic inflammation. As widely reported in the scientific literature, low-grade inflammation is often associated with the development of NCDs (24). However, the observation of inflammation has a lower prevalence compared to blood pressure, glucose, and lipid metabolism (25). In fact, systemic inflammation, which often occurs before hypertension, hyperglycemia, and hyperlipidemia, is a more sensitive indicator for improving metabolism from an early stage (26). In this study, we found that the levels of IL-6, IL-8, TNF-α, and hs-CRP were dramatically reduced, indicating that this multiple-lifestyle approach alleviated inflammation and contributed to further improvement of metabolism. As expected, marginally but statistically significant changes in the lipid profile were observed, that is, a decrease in TG, LDL-C, and apolipoprotein B concentrations and an increase in HDL-C concentrations. A possible explanation is that the study population had mild dyslipidemia, which resulted in no remarkable changes in lipid profiles but indicated an early beneficial trend during the 12-week real-world intervention. In addition, both blood pressure and blood glucose levels decreased slightly, although the differences were not statistically significant. This is probably because metabolism is always affected by interlinked factors, including dietary intake, stress, underlying physiological status, and the interaction between diet and behavior (27). In addition, in terms of blood sugar, time of day or eating window duration also has implications for dietary responses (28), such that eating later induces nocturnal glucose intolerance and reduces fatty acid oxidation and mobilization (29). Food and meal order, including consuming carbohydrates before protein and vegetables in a meal, contributes to elevated glycemic variability (30). Hence, such a relatively short intervention duration may not be sufficient to achieve profound effects.

The encouraging changes in body weight and metabolism were mainly attributable to improvements in diet quality and exercise. The most important and modifiable risk factors for NCDs are unhealthy diets and physical inactivity. A systematic review of 94 cohort studies from the Western Pacific region indicated that dietary intake and other lifestyle factors have a profound influence on the risk of NCDs (31). In our study, participants proactively reduced their intake of rice, wheat, and fried food and increased their intake of fruits and vegetables (F&V), fish, and other aquatic products, eggs, and dairy products. We also observed considerable variability in nutrient intake at the study endpoint. This demonstrated that a reasonable diet, with the guidance of structured health education, resulted in participants adopting lower carbohydrate and higher protein, fat, and dietary fiber intakes compared with the situation before the intervention. In addition, nutrient intakes, except for vitamin A, increased. Each of the dietary behaviors or nutrient intakes that are promising for NCDs is beneficial for health (32) and comprehensively improves metabolism. Of course, it should not be ignored that the reduction in the intake of oil and salt, the protective effects of physical activity, and a supportive environment in the canteen have also provided suggestive evidence of the intervention effect. Notably, the intake of sugar slightly increased during the intervention. Although the average daily added sugar usage in this canteen during the intervention period decreased compared with that before the launch of the Healthy Canteen construction project (data not shown), more publicity and effective means are needed in terms of sugar reduction.

The main advantages of this study are as follows. First, the collective dining ensured the high feasibility of our study, which is helpful to popularize the results of this study in government departments, enterprises, schools, factories, and other collective dining units. Second, we arranged registered dietitians and sports instructors to provide detailed and structured health education to participants throughout the intervention, and the knowledge gained about maintaining a healthy lifestyle is helpful in preventing and controlling NCDs. However, this advantage may be limited to some extent in promoting this study in daily life because of the limited number of registered dietitians. Fortunately, nutrition instructors are required in the construction of nutrition and health canteen, which can not only solve the problem of dietary guidance but also promote the training and employment of nutrition professionals (Additional Files 2, 3). Ultimately, the findings of our study support that the construction of nutrition and health canteens is an important measure with both a high impact and feasibility for closely following national nutrition policies. This would be highly acceptable to the public in China and would have sustained beneficial consequences for being metabolically healthy.

There are some limitations to this study. First, the single-group pre-post design substantially limits causal inference, and a randomized controlled study is needed to verify this conclusion. Second, the sample size was sufficient to detect the primary outcome measure; however, it lacked sufficient capacity to explore the effects on other potentially important indicators. In addition, baseline dietary data were collected and calculated using food frequency questionnaires; however, during the intervention, data were displayed by per capita consumption. Although the per capita daily food intake of participants was calculated using the purchase amount, residual amount, and meal punch card records, consistent methods should be used to confirm these findings in future studies. Moreover, the use of per-protocol (PP) analysis may lead to potential attrition bias and limit the generalizability of the results. We also acknowledge additional limitations such as a short duration and single-center recruitment.

## Conclusion

A nutrition and health canteen strategy-based lifestyle intervention is effective and feasible in improving body weight and metabolism in adults. The results demonstrate that increased food diversity, reduced supply of salt, sugar, and oil, and structured dietary and lifestyle education are beneficial for body weight control and are associated with improved metabolism, which may contribute to the overall prevention and control of NCDs. Further studies, especially randomized controlled trials with longer follow-up periods, are warranted.

## Data Availability

The raw data supporting the conclusions of this article will be made available by the authors, without undue reservation.

## Glossary

NCDs: Non-communicable diseases
CVDs: Cardiovascular and cerebrovascular diseases
MetS: Metabolic syndrome
T2DM: Type 2 diabetes mellitus
CHH: Chinese heart-healthy
DASH: Dietary approaches to stop hypertension
CBDP: Chinese Balanced Diet Pagoda
FFQ: Food Frequency Questionnaire
BMI: Body mass index
WHR: Waist-to-hip Ratio
EDTA: Ethylenediaminetetraacetic acid
FBG: Fasting blood glucose
TC: Total cholesterol
HDL-C: High-density lipoprotein cholesterol
LDL-C: Low-density lipoprotein cholesterol
ApoA1: Apolipoprotein A1
ApoB: Apolipoprotein B
TG: Triglycerides
HOMA-IR: Homeostasis model assessment of insulin resistance
hs-CRP: High-sensitivity C-reactive protein
IL-6: Interleukin-6
IL-8: Interleukin-8
TNF-α: Tumor necrosis factor α
ELISA: Enzyme-linked immunosorbent assay
F&V: Fruits and vegetables
