# Supplementary material for: Nutrition and health canteen-based lifestyle intervention: association with weight management, lipid metabolism regulation, and inflammation alleviation in middle-aged adults
Source: Front Nutr. 2026 Apr 1;13:1754647. doi: 10.3389/fnut.2026.1754647 (PMC13079364; doi:10.3389/fnut.2026.1754647)
Supplement: Supplementary file 1 [file Supplementary_file_1.docx]

Nutrition and Health Canteen-Based Lifestyle Intervention: Association with Weight Management, Lipid Metabolism Regulation, and Inflammation Alleviation in Middle-Aged Adults

**Additional file 1**

Sup. Table 1. Detailed description of the online nutrition health education program*

| Chapters | Title | Content |
| --- | --- | --- |
| Chapter 1 | Lifestyle and NCDs | the importance of nutrition and exercise. |
| Chapter 2 | Rational diet | Reasonable diet means providing a comprehensive and balanced diet. A variety of foods can provide the body with various nutritional needs. |
| Chapter 3 | Three reductions and three health (part 1) | "Three reductions" related content, that is, reduce the intake of lipids, salt and sugar. |
| Chapter 4 | Three reductions and three health (part 2) | "Three health" related content, that is, achieve a healthy weight, bone and oral health. |
| Chapter 5 | Dietary guidelines | Read the latest CFGP (2022) Eight Guidelines for a Balanced diet. |
| Chapter 6 | Sports concept | Eat dynamic balance, moderate exercise, maintain a healthy weight; The index of body mass index, which is used to judge healthy weight, is also introduced. |
| Chapter 7 | High quality protein | How to eat more dairy and soy to increase sources of calcium, high-quality protein and micronutrients. |
| Chapter 8 | Nutrition label | Learn to read the ingredient list and nutrition label on food packaging correctly, and buy products to avoid the merchant trap. |
| Chapter 9 | Drink enough water | Provide scientific drinking methods for people with different characteristics and different physical conditions. |
| Chapter 10 | Control sugar and salt | This paper introduces WHO's recommended intake of sugar and salt, and reminds residents of the daily risk of excessive intake of sugar and salt. |
| Chapter 11 | Nutrition-related disease | Various nutrients due to the excessive or lack of content in the body, or each other imbalance caused by a class of nutritional metabolic disorders. |
| Chapter 12 | Summary | Summary of knowledge points. |

* Mainly based on the Dietary Guidelines for Chinese Residents (2022).

Sup. Table 2. Detailed description of the online exercise health education program

| Twelve-week Training Cycle Plan* | | | |
| --- | --- | --- | --- |
| Training Periods | Basic Adaptation Phase | Intensive Enhancement Phase | Consolidation and Maintenance Phase |
| Key Objectives | Learning and mastering fundamental movements. | Building a correct movement pattern and fundamental skills by High-Intensity Interval Training (HIIT). | Utilizing a combination of diverse training methods and significantly enhance training load. |
| Training Goals | Initial improvements in aerobic capacity, muscle strength, and core stability. | Observable enhancements in aerobic capacity, muscle strength, and core stability. | Significant progress in muscle strength, muscular endurance, and aerobic fitness. |
| Training Principles | Low intensity  Long duration  Low training loads | Moderate intensity  Moderate duration  Medium training loads | High intensity  Moderate duration  High training loads |
| Training Load Distribution | 50%-60% | 60%-70% | 70%-80% |
| Overall Schedule | The training plan adheres to the principles of specificity, appropriate load management, and gradual progression to ensure sustainable and measurable improvements.   1. Maintain daily physical activity and increase the amount of activity. 2. Do at least 2 days of strength exercises per week.  - 20 minutes of warm-up exercises. - 50 minutes of core training activities. - 20 minutes of stretching and relaxation. | | |

* Based on the Chinese population physical activity guidelines (2021).

Sup. Table 3. Self-reported physical activity levels at baseline.

|  | N | Ratio（%） |
| --- | --- | --- |
| Frequency |  |  |
| None | 10 | 10.31 |
| 1-2 times/week | 53 | 54.64 |
| 3-4 times/week | 20 | 20.62 |
| > 4 times/week | 14 | 14.43 |
| Time duration |  |  |
| < 30 min/time | 24 | 24.74 |
| 30-60 min/time | 60 | 61.86 |
| 60-120 min/time | 13 | 13.40 |
| > 120 min/time | 0 | 0 |
| Types of exercise |  |  |
| Walking | 74 | 76.29 |
| Jogging | 36 | 37.11 |
| Ball games | 36 | 37.11 |
| Rope skipping | 6 | 6.19 |
| Aerobic exercise | 16 | 16.49 |
| Swimming | 11 | 11.34 |
| Cycling | 5 | 5.15 |
| Instrument training | 19 | 19.59 |

Sup. Table 4. Detailed consumption of various foods during the intervention period.

| Food category | Total consumption (kg) | Per capita daily consumption (g/d) |
| --- | --- | --- |
| Rice | 488.53 | 69.76 |
| Wheat | 724.18 | 103.41 |
| Coarse cereals | 440.21 | 62.86 |
| Tubers | 124.93 | 17.84 |
| Fresh vegetables | 1091.07 | 155.80 |
| Fresh fruits | 891.13 | 127.25 |
| Pork | 436.43 | 62.32 |
| Beef and mutton | 371.02 | 52.98 |
| Poultry | 252.74 | 36.09 |
| Fish | 158.90 | 22.69 |
| Other aquatic products | 109.74 | 15.67 |
| Eggs | 352.81 | 50.38 |
| Milk | 700.44 | 100.02 |
| Yogurt | 933.78 | 133.34 |
| Soybeans | 56.16 | 8.02 |
| Soy products | 133.2 | 19.02 |
| Nuts | 58.69 | 8.38 |
| Fried foods | 548.62 | 78.34 |
| Oil | 205.00 | 29.27 |
| Salt | 41.25 | 5.89 |
| Sugar | 199.00 | 27.13 |

Sup. Table 5. Effects of intervention on metabolism

|  | Before | after | *P-*value | *P_FDR_*^a^ |
| --- | --- | --- | --- | --- |
| Anthropometric data (n=85) |  |  |  |  |
| Weight (kg) | 74.00 (61.05-81.6) | 72.35 (58.83-80.15) | **<0.001** | **0.025** |
| BMI (kg/m^2^) | 23.86±3.14 | 23.37±3.18 | **<0.001** | **0.016** |
| WHR | 0.89 (0.85-0.91) | 0.87 (0.82-0.9) | **0.013** | 0.052 |
| Body fat mass (kg) | 17.85±5.43 | 17.82±5.02 | 0.949 | 0.978 |
| Body fat percentage (%) | 26.48±5.30 | 25.80±5.41 | 0.177 | 0.225 |
| Muscle content (kg) | 26.60 (22.20-32.38) | 30.40 (23.70-33.50) | **0.001** | **0.032** |
| Basal metabolism (kcal) | 1411.50 (1255.50-1609.25) | 1545.00 (1308.00-1648.00) | **0.003** | **0.024** |
| Body moisture content (kg) | 40.00 (31.80-43.50) | 35.45 (29.98-42.03) | **0.004** | **0.033** |
| Protein (kg) | 10.70 (8.50-11.80) | 9.50 (8.00-11.43) | **0.001** | **0.012** |
| Inorganic salt (kg) | 3.46±0.59 | 3.39±0.59 | **0.006** | **0.027** |
| Pulse (Times/min) | 81.02±12.82 | 77.98±11.85 | 0.120 | 0.337 |
| Blood pressure (n=85) |  |  |  |  |
| SBP (mmHg) | 114.50 (103.00-124.25) | 110.00 (102.00-125.00) | 0.560 | 0.765 |
| DBP (mmHg) | 74.00 (64.00-81.00) | 70.00 (65.00-79.00) | 0.581 | 0.813 |
| Blood glucose (n=72) |  |  |  |  |
| FBG (mmol/L) | 4.96 (4.66-5.29) | 4.76 (4.48-5.13) | 0.590 | 0.766 |
| Insulin (μIU/mL) | 8.29 (6.69-10.71) | 9.18 (6.86-11.90) | **0.036** | 0.092 |
| HOMA-IR | 1.98±1.16 | 2.29±1.56 | 0.102 | 0.155 |
| Blood lipids (n=72) |  |  |  |  |
| TC (mmol/L) | 4.90±0.80 | 4.80±0.79 | 0.093 | 0.103 |
| TG (mmol/L) | 1.17 (0.78-1.70) | 1.12 (0.74-1.87) | **0.030** | **0.047** |
| HDL-C (mmol/L) | 1.32±0.33 | 1.37±0.34 | **0.032** | **0.044** |
| LDL-C (mmol/L) | 2.76±0.70 | 2.58±0.62 | **<0.001** | **0.041** |
| Apolipoprotein A1 (g/L) | 1.47±0.16 | 1.47±0.16 | 0.970 | 0.977 |
| Apolipoprotein B (g/L) | 0.83±0.20 | 0.78±0.18 | **<0.001** | **0.045** |

^a^ *P_FDR_* means the P value after multiple comparison using the false discovery rate (FDR) method.

**Additional file 2**

**Construction Guide for Nutritious and Healthy Canteens program (the quotation of content related to this study).**

These guidelines are formulated in accordance with the requirements of "Healthy China Action (2019-2030)" and "National Nutrition Plan (2017-2030)" to guide and standardize the construction of nutritious and healthy cafeterias.

These guidelines are applicable to food service operators whose main business model is institutional cafeterias (staff canteens). Cafeterias in primary schools, high schools, and colleges/universities may refer to these guidelines for implementation.

**Article 6 Nutritional Health Education**

1. Various forms of publicity should be adopted to promote knowledge about balanced diet,“low salt, oil, and sugar, prevention and control of nutrition-related chronic diseases, infectious disease prevention, food conservation policies, and scientific knowledge to create a nutritious and healthy dining atmosphere. These include:

- Displaying, hanging, or placing materials and playing videos in prominent locations;
- Promoting the "Chinese Dietary Guidelines" and the Chinese Food Guide Pagoda, as well as providing recommendations for daily and three-meal intake of energy, fats, and other nutrients;
- Providing freely accessible promotional materials such as booklets, brochures, and flyers in or near the cafeteria.

1. The cafeteria should take the lead in organizing nutrition-related promotional activities, including nutrition and health lectures, knowledge quizzes, and culinary competitions, no less than twice per year.
2. The cafeteria is encouraged to actively disseminate nutrition and health knowledge, and solicit opinions and suggestions from diners.

**Article 7 Food Service and Culinary Requirements**

1. Food categories should meet the recommendations of the 'Chinese Dietary Guidelines.' Each meal menu must include at least 3 food categories (not including seasonings and vegetable oils), and foods within the same category may be substituted. Requirements for food categories and varieties in the menu：

1) Grains, Tubers, and Legumes

· Minimum of 5 varieties per week

· Maintain a balance between whole and refined grains

1. Vegetables and Fruits

· Minimum of 10 varieties of fresh vegetables per week

· Dark-colored vegetables should comprise over 50% of total vegetable offerings

· Fruit service is encouraged

1. Protein Sources

· Minimum of 5 varieties per week

· Priority should be given to: Aquatic products，Eggs，Poultry

· When serving meat, lean cuts should be prioritized

1. Dairy and Soy Products

· Minimum of 5 varieties per week

1. Vegetable Oils

· Use a diverse selection of vegetable oils

· Minimize or avoid hydrogenated vegetable oils

· Public disclosure required if hydrogenated oils are used

2. Cooking Methods and Nutritional Principles Food preparation methods should adhere to nutritional and health principles:

1) Preferred Cooking Methods

· Prioritize techniques that minimize nutrient loss

· Maintain natural food flavors

· Focus on healthier cooking approaches

1. Limited Cooking Methods

· Minimize use of:

· Deep frying

· Pan frying

· Smoking

· Grilling/roasting

1. Innovation in Healthy Cooking

· Encourage development of new healthy cooking techniques

· Preserve distinctive flavors while reducing:

· Salt

· Oil

· Sugar

· Seasonings containing these ingredients

1. Overall Objectives

· Maintain nutritional value

· Preserve authentic taste

· Reduce unhealthy ingredients

· Promote healthier cooking innovations

3. Dietary Control Guidelines

1) Menu Requirements

· Prioritize dishes that are: Low in salt，Low in oil，Low in sugar

· Reduce offerings of dishes high in these ingredients

1. Dining Area Policy

· Salt and sugar dispensers should not be placed in dining areas

1. Nutritional Meal Planning Requirements
2. Menu Planning

· Develop balanced nutritional meal plans

· Avoid menu repetition within the same week for cafeterias serving set meals or individual portions

1. Self-Service Cafeteria Guidelines

Encouraged to provide standardized meal sets considering:

· Seasonal and weather conditions

· Physical intensity of employees' work

· Age and gender demographics

· Daily energy requirements

· Essential nutrient needs

1. Meal Planning Considerations

Standardized meals should:

· Meet recommended energy requirements per meal

· Provide adequate essential nutrients

· Accommodate workplace-specific needs

· Ensure nutritional balance

1. All cafeterias must prominently display nutritional information. Those offering set meals or individual portions are required to show both portion sizes and nutritional content. While self-service facilities must display nutritional information, they are also encouraged to provide portion guidance through detailed menu information.
2. To better serve diverse health needs, cafeterias are encouraged to offer specialized meal options. This includes providing tailored nutritional meals for individuals managing obesity or nutrition-related conditions (This provision excludes therapeutic meals designed for hospital patients).
3. The implementation of smart systems in cafeterias is encouraged to facilitate both meal planning and dining guidance.

**Article 8 Creating a Nutritious and Healthy Environment**

1. In prominent places in the canteen, publicity materials on healthy eating are posted, which can include, but are not limited to, the "Dietary Guidelines for Chinese Residents" and the Chinese Residents' Balanced Diet Pagoda, reasonable diet, "reducing salt, oil and sugar", prevention and treatment of chronic diseases related to nutrition, prevention and control of infectious diseases, food saving and other policies and popular science knowledge.

2. Provide nutrition and health promotion materials that are freely available in the canteen or nearby places, such as brochures, leaflets, single pages, etc.

1. Set up a "nutrition and health corner", place height, weight measuring instruments, body mass index (BMI) test plates, etc., and ensure that the equipment can be used normally.

**Article 9 Personnel training and assessment**

1. Provide full-time (or part-time) nutritionists / nutrition instructors with relevant qualification documents.

1. Provide nutrition and health knowledge and skills training for staff.
2. Chefs are required to receive culinary skills training no less than twice a year, focusing on dishes with low salt, less oil, and low sugar.

**Additional file 3**

**Sample daily menu for a nutritious and healthy canteen**

| Category | Contents |
| --- | --- |
| Breakfast | Cold Dishes : Shredded Chicken Salad, Fried peanuts, Mushroom and Onion Salad  Hot Dishes: Minced Pork with Fresh Green Beans, Stir-fried Chinese Yam with Bamboo Shoots, Braised Tofu with Tomatoes, Dry-fried Green Vegetables, Home-style Shredded Potatoes, Scrambled Eggs with Green Peppers  Pastries: Steamed Buns, Steamed rolls, Boiled Eggs, Pork and Vermicelli Buns, Chinese Fried Dough (Youtiao), Chicken Wonton Soup  Beverages: Millet Porridge, Red Bean Porridge, Milk, Soybean Milk |
| Lunch | Cold Dishes: Five-spice Pork Knuckle, Garlic Mustard Greens, Mixed Vegetable Salad  Hot Dishes: Braised Pork with Taro, Braised Chicken with Brown Sauce, Three-color Shrimp, Stir-fried Pork, Braised Tofu, Stir-fried Tomatoes with Eggs, Stir-fried Chinese Flowering Cabbage, Shredded Cabbage  Pastries: Steamed Rice, Red Bean Rice, Steamed Buns, Twisted Rolls, Xi'an Roujiamo (Chinese Hamburger), Steamed Sweet Potato, Handmade Liangpi (Cold Rice Noodles), Traditional Zijuan Pancake, Wugong Qihua Noodles  Beverages: Fresh Meatball Soup, Beauty Tonic Soup, Yogurt  Fruits: Honeydew Melon, Red Dragon Fruit |
| Dinner | Cold Dishes: Deep-fried Yellow Croaker, Celery with Bean Curd Sheets, Spinach with Garlic  Hot Dishes: Crispy Diced Pork, Chicken Slices with Two Kinds of Mushrooms, Shredded Pork with Green Peppers, Sour and Spicy Baby Cabbage, Mushrooms with Green Vegetables, Broccoli with Minced Garlic  Staples & Snacks: Steamed Rice, Twisted Rolls, Steamed Buns, Radish Pancake, Purple Rice Cake, Steamed Pumpkin  Beverages: Sweet Corn Soup, Red Date Rice Porridge, Lantian Maohualuo (Traditional Noodle Soup)  Fruits: Mandarin Orange, Banana |
